# fMRI network correlates of predisposing risk factors for delirium: A cross-sectional study

**DOI:** 10.1016/j.nicl.2020.102347

**Published:** 2020-07-15

**Authors:** S.J.T. van Montfort, A.J.C. Slooter, I.M.J. Kant, R.R. van der Leur, C. Spies, J. de Bresser, T.D. Witkamp, J. Hendrikse, E. van Dellen

**Affiliations:** a Department of Intensive Care Medicine and UMC Utrecht Brain Center, University Medical Center Utrecht, Utrecht University, The Netherlands; b Faculty of Medicine, University of Utrecht, The Netherlands; c Department of Anaesthesiology, Charité Universitätsmedizin Berlin, Freie Universität Berlin, Humboldt-Universität zu Berlin and Berlin Institute of Health, Berlin, Germany; d Department of Radiology, Leiden University Medical Center, The Netherlands; e Department of Radiology and UMC Utrecht Brain Center, University Medical Center Utrecht, Utrecht University, The Netherlands; f Department of Psychiatry and UMC Utrecht Brain Center, University Medical Center Utrecht, Utrecht University, The Netherlands

**Keywords:** Delirium, Risk factors, Brain networks, Graph theory, Functional connectivity, Aging

## Abstract

Delirium, the clinical expression of acute encephalopathy, is a common neuropsychiatric syndrome that is related to poor outcomes, such as long-term cognitive impairment. Disturbances of functional brain networks are hypothesized to predispose for delirium. The aim of this study in non-delirious elderly individuals was to investigate whether predisposing risk factors for delirium are associated with fMRI network characteristics that have been observed during delirium. As predisposing risk factors, we studied age, alcohol misuse, cognitive impairment, depression, functional impairment, history of transient ischemic attack or stroke, and physical status. In this multicenter study, we included 554 subjects and analyzed resting-state fMRI data from 222 elderly subjects (63% male, age range: 65–85 year) after rigorous motion correction. Functional network characteristics were analyzed and based on the minimum spanning tree (MST). Global functional connectivity strength, network efficiency (MST diameter) and network integration (MST leaf fraction) were analyzed, as these measures were altered during delirium in previous studies. Linear regression analyses were used to investigate the relation between predisposing delirium risk factors and delirium-related fMRI characteristics, adjusted for confounding and multiple testing. Predisposing risk factors for delirium were not associated with delirium-related fMRI network characteristics. Older age within our elderly cohort was related to global functional connectivity strength (β = 0.182, p < 0.05), but in the opposite direction than hypothesized. Delirium-related functional network impairments can therefore not be considered as the common mechanism for predisposition for delirium.

## Highlights

• Predisposing risk is not associated with delirium-related fMRI characteristics.
• Older age within an elderly cohort is related to higher functional connectivity strength.
• This relation is in opposite direction than hypothesized.
• The onset of delirium may reflect new functional network impairments.

## 1 Introduction

Delirium is an acute and common neuropsychiatric syndrome, affecting 10–50% of the hospitalized elderly patients (Marcantonio, 2017). The syndrome is by definition a consequence of one or more medical conditions, and predominantly characterized by a disturbance in attention and awareness, with additional cognitive deficits (American Psychiatric Association, 2013). Delirium is a burden for patients and related to poor outcomes, such as long-term cognitive impairment (Marcantonio, 2017). From an etiological perspective, risk factors for delirium can be distinguished into predisposing factors (i.e. baseline vulnerability for delirium, for example older age), and precipitating factors (i.e. acute changes that trigger the syndrome, for example an infectious disease) (Inouye et al., 2014). The development of delirium is usually the result of interaction of several different risk factors (Inouye et al., 2014, Marcantonio, 2017, Zaal et al., 2015).

While such etiological models aim to understand the underlying biological mechanism of risk for delirium, prediction models aim to predict the occurrence of delirium with a certain accuracy irrespective of mechanistic assumptions of causality. Etiological models on delirium have shown a range of relative risk values of predisposing risk factors for delirium (relative risk scores of dementia: 2.3–4.7; cognitive impairment: 1.3–4.2; history of delirium: 3, functional impairment 2.5–4.0; visual impairment: 1.1–3.5; hearing impairment: 1.3; severity of illness or physical status: 1.1–5.6; depression: 1.2–3.2; history of transient ischemic attack or stroke: 1.6; alcohol misuse: 1.4–5.7; older age: 1.1–6.6) (Inouye et al., 2014). Relative risk values or etiological fractions of predisposing risk factors are difficult to quantify as they probably consist of an interaction between predisposition and precipitating events (which may even be non-linear) (van Montfort et al., 2019).

The neural substrate of predisposition for delirium remains poorly understood, and is hypothesized to reflect the cumulative effects of aging and physical, cognitive and psychological frailty. Focusing on the shared biological characteristics of predisposing risk factors allows us to increase our understanding of the risk profile of delirium before acute changes, (such as an infectious disease or trauma), occur.

It has been hypothesized that delirium is a disconnection syndrome, caused by the breakdown of functional brain networks (Sanders, 2011, van Dellen et al., 2014, van Montfort et al., 2019, Young, 2017). The functional network may represent the communication between different brain regions (Bassett and Sporns, 2017). Brain network organization can be characterized based on functional connectivity maps, representing the statistical interdependencies of time-series recorded from different brain areas, for example measured with imaging techniques such as functional magnetic resonance imaging (fMRI) (Aertsen et al., 1989, van den Heuvel and Hulshoff Pol, 2010). It has been shown that during delirium, the network was less efficient organized and less integrated (Numan et al., 2017, van Dellen et al., 2014, van Montfort et al., 2018). Although disturbances between several brain regions have been suggested during delirium, the functional connectivity between two specific regions that could be involved in cognition, attention or consciousness, was found to be altered during delirium in two independent studies, i.e. between the posterior cingulate cortex (PCC) and the dorsolateral prefrontal cortex (DLPFC) (Choi et al., 2012, Oh et al., 2019). In addition, a recent review evaluating network studies on delirium and its risk factors suggested that predisposing delirium risk factors are generally associated with decreased global functional connectivity strength (van Montfort et al., 2019). Functional network impairments may therefore be a common mechanism in the pathophysiology of delirium and a possible biological pathway towards vulnerability for delirium. In this way, vulnerability may correspond to a lower threshold for a transition from a healthy state towards encephalopathy with disturbed brain activity that manifests as delirium (Slooter et al., 2020).

Investigating the integrated effect of delirium risk factors on the functional network may support this hypothesis and may lead to a unified understanding of delirium vulnerability associated with a variety of heterogeneous factors. However, a previous study did not show strong relationships between electroencephalography (EEG) (network) characteristics and predisposing risk factors for delirium (van Montfort et al., 2020). fMRI has a superior spatial resolution compared to EEG, and could be used to integrate functional brain network analysis with neuroanatomical information, such as functional connectivity between specific regions. Analysis of the association between delirium risk factors and fMRI networks may therefore provide important additional information on altered network organization as a common mechanism to explain vulnerability for delirium. Accordingly, we note that rather than studying delirium itself, we specifically studied risk factors for delirium. The aim of the present study was to evaluate the effect of predisposing delirium risk factors on fMRI network characteristics in an elderly cohort. It was hypothesized that predisposing risk factors for delirium, separate or combined, are associated with delirium-related fMRI network characteristics, i.e. decreased functional connectivity strength, decreased network efficiency and decreased network integration. As a secondary analysis, we evaluated the effect of predisposing delirium risk factors on the regional connection between the PCC and the DLPFC. Although the etiology of delirium is complex and multifactorial, the exact weight or relative risk of independent risk factors is unknown. The inclusion of risk factors was based on a recent high quality review on delirium (Inouye et al., 2014).

## 2 Methods

### 2.1 Study design and population

This study is part of the *Biomarker Development for Postoperative Cognitive Impairment in the Elderly* (BioCog) project at the University Medical Center (UMC) Utrecht and Charité Hospital at Berlin (Winterer et al., 2018). In the current cross-sectional sub-study, elderly individuals were included, who were non-hospitalized participants scheduled to undergo elective surgery (i.e. orthopedic-, cardiac-, gastro-intestinal-, maxillofacial- or otorhinolaryngologic surgery), as well as participants that were recruited via a local general practitioner. Inclusion criteria were European ancestry, -age of 65 year or over, and signed informed consent for the study. Participants with one or more of the following characteristics were excluded: a life expectancy shorter than a year; an indication for (early) dementia as indicated with a score of 23 or lower on the Mini Mental State Examination (MMSE) (Folstein et al., 1985); missing fMRI data. fMRI measurements and clinical assessments were performed on the same day.

### 2.2 Clinical assessment

Risk factors evaluated in this study were based on a high quality review (Inouye et al., 2014). We were not able to investigate all risk factors described in the review, i.e. participants with dementia, history of delirium and unsolved hearing or visual impairment were unavailable, comorbidity was not measured within this study.

#### 2.2.1 Age

To determine age, the medical records of the participants were used.

#### 2.2.2 Alcohol misuse

To define alcohol misuse the self-reported Alcohol Use Disorders Identification Test (Audit) was used. The Audit is a validated questionnaire of 10 items that assesses alcohol consumption, drinking behaviors, and alcohol-related problems (Bohn et al., 1995, Reinert and Allen, 2007). A cut-off value of 8 points was used to determine alcohol misuse (Babor et al., 2001).

#### 2.2.3 Cognitive impairment

To define cognitive impairment, the total score on the MMSE was used (Folstein et al., 1985) and studied as continuous variable.

#### 2.2.4 Depression

To define depression, the Geriatric Depression Scale (GDS) with 15 items was used (Yesavage et al., 1982, Yesavage and Sheikh, 1986). A score of 6 was used as cut-off to determine depression.

#### 2.2.5 Functional impairment

Functional impairment was defined with the validated Barthel Index following the Hamburg classification manual (Collin et al., 1988, Lübke et al., 2004, Mahoney and Barthel, 1965). The continuous outcome measure was the total score (0–100), where the maximum score of 100 indicates fully independent functional ability.

#### 2.2.6 History of transient ischemic attack or stroke

To determine history of transient ischemic attack (TIA) or stroke, the medical records of the participants were used. If this information was not available, participants were asked whether they had experienced a TIA or stroke. If either or both were positive, this risk factor was considered present. In addition, cortical, subcortical and lacunar infarcts, were scored based on the STRIVE criteria (Wardlaw et al., 2013) by a neuroradiologists (TW or JB) by use of the T1-weighted, the fluid-attenuated inversion recovery (FLAIR) sequence and the diffusion-weighted image (DWI). The final classification of TIA or stroke was based on all available information.

#### 2.2.7 Physical status

Physical status was defined using the American Society of Anesthesiologists (ASA) classification. The validated ASA score is widely used for the assessment of preoperative physical status (Aronson et al., 2003, Sankar et al., 2014), ranging from I. healthy; II. mild systematic disease; III severe systematic disease that is not incapacitating; IV. incapacitating systematic disease that is a constant threat to life; to V. moribund status, not expected to survive for 24 h without surgery (Owens et al., 1978). We studied this measure dichotomized, where an ASA-score of I was classified as healthy and an ASA-score of II or higher as unhealthy.

#### 2.2.8 Estimated intelligence coefficient (IQ)

The validated reading test for adults ‘Nederlandse leestest voor volwassenen’ (NLV) for the Dutch subjects or the ‘Mehrfachwahl-Wortschatz-Intelligenztest’ (MWT-A) for the German subjects was used to estimate premorbid IQ (Lehrl et al., 1995, Mulder et al., 2012). The raw scores were converted to an estimated IQ score.

### 2.3 Image processing

#### 2.3.1 MRI scans

Imaging was performed on a 3 T Achieva (Philips Medical Systems, Best, the Netherlands) scanner in Utrecht and on a 3 T TrioTim (Siemens Healthineers, Erlangen, Germany) scanner in Berlin. For the structural scan, a T1-weighted 3D Turbo Field Echo (TFE) image or a T1-weighted Magnetization Prepared Rapid Gradient Echo (MPRAGE) image was made, respectively. The sequence parameters of the T1 TFE were: TR = 7.9 ms, TE = 4.5 ms, flip angle = 8°, 192 sagittal slices, voxel size 1 × 1 × 1 mm. The sequence parameters of the T1 MPRAGE were: TR = 2500 ms, TE = 4.77 ms, flip angle = 7°, 192 sagittal slices, voxel size 1 × 1 × 1 mm. For the resting-state blood-oxygen-level dependent (BOLD) fMRI (rs-fMRI) scan, a T2*-weighted gradient-echo – echoplanar imaging (GE-EPI) image was used with the following sequence parameters: TR = 2000 ms, TE = 30 ms, flip angle = 78°, 32 transversal slices, voxel size 3 × 3 × 3, 75 mm, 238 volumes in 7 min and 55 s. The rs-fMRI was made in a dark room and participants were asked to close their eyes and to stay awake. For visual inspection of brain infarcts a FLAIR (TR = 4800, TE = 125, inversion time = 1650 ms (Utrecht); TR = 4800, TE = 388, inversion time = 1800 ms (Berlin) and DWI (voxel size = 0.96 × 1.19 × 4.00 mm^3,^ TR = 3294, TE = 68 ms (Utrecht only)) was used.

#### 2.3.2 Preprocessing

Image preprocessing was performed using the FMRIB’s Software Library (FSL) (Jenkinson et al., 2012, Smith et al., 2004, Woolrich et al., 2009). The brain was automatically extracted from the T1-weighted scan (Smith, 2002). Time series were motion corrected with MCFLIRT (Jenkinson et al., 2002, Jenkinson and Smith, 2001). Participants with a mean relative displacement larger than 0.2 mm were excluded (Power et al., 2012). It has been recognized that motion during the fMRI measurement can induce systematically bias inference, therefore additional motion correction is necessary (Ciric et al., 2017, Power et al., 2015, Power et al., 2012, Pruim et al., 2015). Volumes that exceeded the threshold of 0.2 mm framewise displacement (Power et al., 2014) were removed and a regression analysis with 36 motion components was done. Motion components were: three voxel-wise displacement parameters and their white matter, cerebrospinal fluid, global time courses, and the quadrates, temporal derivatives and quadrates of the derivatives of these six parameters (Satterthwaite et al., 2013). Average time series from the cerebral spinal fluid, the white matter and grey matter intensities were determined after tissue segmentation with the FMRIB's Automated Segmentation Tool (FAST) (Zhang et al., 2001). A band-pass filter (0.01–0.08 Hz) was applied (Satterthwaite et al., 2013). The functional scan was registered to the high-resolution anatomical image by using rigid registration. The anatomical scan was subsequently matched with the Montreal Neurological Institute (MNI) 152 T1-weighted 2 mm image in standard space with affine registration. Functional scans were slice-time corrected and spatial smoothed to reduce noise (5 mm full-width-half-maximum). The first 15 volumes were deleted to ensure stabilized magnetization. If the remaining data was less than 240 s, the subject was excluded from further analysis (Birn et al., 2013).

#### 2.3.3 Connectivity and network analysis

**Fig. 1:**
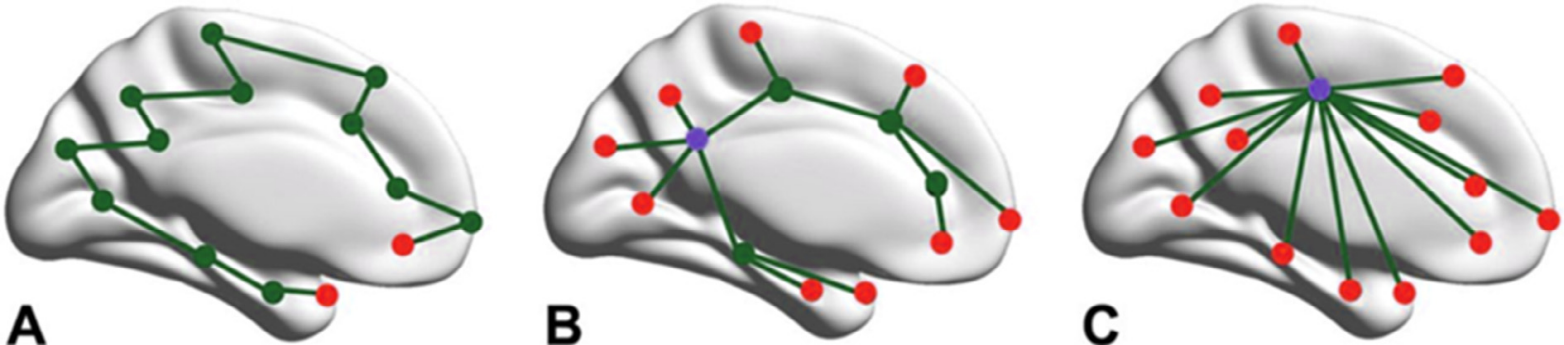
Schematic representation of the minimum spanning tree. Minimum spanning trees can conceptually range between a path-like tree (a less efficient and sparsely integrated network) and a star-like tree (an highly efficient and highly integrated network). Diameter is the length of the path between the two nodes that are furthest apart, and a measure for network efficiency. Leaf fraction is the fraction of leaf nodes (red), i.e. nodes that only have one edge, and therefore a measure of network integration. (A) Line-like network: few leaf nodes and a long diameter, (B) hierarchical tree structure: conceptually optimal topology, (C) star-like network: many leaf nodes + short diameter, central node (purple) will easily be overloaded with information. (For interpretation of the references to colour in this figure legend, the reader is referred to the web version of this article.)

We selected 264 regions putative functional areas that cover the cortical and subcortical brain regions (Power et al., 2011). To estimate 264 regional mean time series, voxel time series within each region were averaged. Functional connectivity was subsequently calculated between all time series pairs using Pearson’s correlations, resulting in a 264 × 264 functional connectivity matrix for every participant. Minimum spanning tree (MST) network backbones were extracted using Kruskal’s algorithm (MATLAB, version R2016b) (Kruskal, 1956). Only positive correlations were taken into account as a result of the MST analysis, thus avoiding the problematic interpretation of negative BOLD correlations (Stam et al., 2014, Tewarie et al., 2015). The MST can be considered as the backbone of the original network, connecting all regions without forming loops (Stam et al., 2014, Tewarie et al., 2015), which allows a relatively unbiased comparison with another network with the same number of regions (Stam et al., 2014, Tewarie et al., 2015, van Diessen et al., 2015) (Fig. 1). Correlation values of the connectivity matrix were ranked and the highest value was included as the first MST connection using Kruskal’s algorithm (Kruskal, 1956). The second highest value was then added as an MST connection, until all 264 regions were connected. If adding a connection would result in a loop or triangle, this connection was discarded and the next value was evaluated. Note that formally, a maximum spanning tree was constructed; the highest connectivity values were used to construct the MST as these connections were expected to reflect communication with minimal cost. We refer to the minimum spanning tree or MST throughout this manuscript to be consistent with previous literature using this approach. Since it was previously shown that global functional network connectivity, network efficiency, network organization and the regional connectivity between the PCC and the DLPFC were altered in relation to (risk for) delirium, these outcomes were evaluated in our study (Choi et al., 2012, Numan et al., 2017, Oh et al., 2019, van Dellen et al., 2014, van Montfort et al., 2018).

#### 2.3.4 Global functional connectivity strength

For each participant, global functional connectivity strength was calculated by averaging the connectivity values of the connections in the MST (Fig. 1).

#### 2.3.5 Network efficiency (MST diameter)

The MST diameter was used to assess network efficiency (Fig. 1). It describes the number of edges connecting the most remote nodes in the MST and gives an indication of the efficiency of global network organization (Stam et al., 2014, Tewarie et al., 2015). A low diameter describes a network in which information is efficiently processed between remote brain regions (Stam et al., 2014, Tewarie et al., 2015, van Dellen et al., 2016).

#### 2.3.6 Network integration (MST leaf fraction)

The MST leaf fraction was used to estimate network integration (Fig. 1). It describes the proportion of regions with a degree of one, i.e. regions that are connected to only one other region (Stam et al., 2014, Tewarie et al., 2015). A large leaf fraction describes a network that is highly integrated (Stam et al., 2014, Tewarie et al., 2015, van Dellen et al., 2016).

#### 2.3.7 Regional functional connectivity between the PCC and the DLPFC

The PCC was defined as the region centered at coordinates (MNI x/y/z): –11/–56/16 (Power atlas region #77), −3/-49/13 (Power atlas region #78) and 11/-54/17 (Power atlas region #82) (Choi et al., 2012, Oh et al., 2019, Power et al., 2011). The DLPFC left was defined as the region centered at coordinates (MNI x/y/z): −42/38/21 (Power atlas region #167) and −34/55/4 (Power atlas region #176) (Choi et al., 2012, Oh et al., 2019, Power et al., 2011). The DLPFC right was defined as the region centered at coordinates (MNI x/y/z): 38/43/15 (Power atlas region #168) and 40/18/40 (Power atlas region #175) (Choi et al., 2012, Oh et al., 2019, Power et al., 2011). The connection between the PCC and the left or right DLPFC was calculated for each participant using Pearson’s correlations between the mean time series of the regions.

### 2.4 Statistical analysis

Variables that were not normally distributed, were log transformed for all analyses. The association of the individual risk factors on the five outcome measures (i.e. global functional connectivity strength, MST diameter, MST leaf fraction, PCC-DLPFC left and PCC-DLPFC right connectivity strength) were analyzed in separate linear regression models. As age, gender and IQ can be considered as confounders for delirium and network outcomes, we adjusted for center, age (if age was not the determinant), gender and IQ in the analyses (Marcantonio, 2017, Otte et al., 2015, Stumme et al., 2020, van Dellen et al., 2018). The associations of all seven risk factors combined on the five outcome measures, adjusted for center, gender and IQ, were studied with three different multivariable linear regression models. To avoid the report of false negative findings, additional, exploratory analyses were performed on the extremes of the distribution of a possible indicator (highest versus lowest quintile).

To control for multiple testing a False Discovery Rate (FDR) correction was applied using the Benjamini and Hochberg method (Benjamini and Hochberg, 1995, Genovese et al., 2002). After FDR correction, a corrected p-value below 0.05 was considered statistically significant (Benjamini and Hochberg, 1995). Statistical analyses were performed in IBM SPSS Statistics version 21.

## 3 Results

### 3.1 Demographics

In this study, 554 participants were eligible (Table 1). From the eligible participants, 17 were excluded due to discontinuation of the fMRI measurement, 251 were excluded due to insufficient quality of the fMRI scan because of motion, and 64 were excluded due to missing clinical data. Our total sample therefore consisted of 222 participants with complete data on all clinical variables, of whom 182 were non-hospitalized participants scheduled to undergo elective surgery and 40 were participants recruited via a local general practitioner. Table 2 shows the demographics and risk factors for delirium of the included participants used for analyses. Compared to the total cohort, our study population contained more subjects from the center Utrecht, more males and more subjects that had a history of TIA or stroke, was younger and more healthy (Table 1). No correlation was found between relative motion and global functional connectivity strength, MST diameter or MST leaf fraction (Supplementary Information Figure S1 + Figure S2).

**Table 1:** Demographics and risk factors for delirium of the eligible subjects and the total included sample.

	Cohort (eligible subjects; N = 554)	Included subjects (N = 222)	Statistics
Center			
Berlin, n (%)	322 (58)	67 (30)	χ^2^ = 70.53, p = 0.000*
Utrecht, n (%)	232 (42)	155 (70)	
Male, n (%)	338 (61)	140 (63)	χ^2^ = 6.75, p = 0.009*
IQ, mean ± SD	105 ± 12.7	105 ± 12.2	t = 0.00, p = 1.000
Age in years, mean ± SD	72.1 ± 5.0	71.2 ± 4.9	t = 2.30, p = 0.022*
Alcohol misuse			χ^2^ = 0.00, p = 0.975
Yes, n (%)	25 (5)	11 (5)	
No, n (%)	485 (95)	211 (95)	
MMSE (cognitive impairment)	28.6 ± 1.4	28.7 ± 1.4	t = -0.90, p = 0.369
GDS (depression)			χ^2^ = 0.00, p = 0.975
Yes, n (%)	24 (5)	10 (5)	
No, n (%)	447 (95)	212 (95)	
BI (functional impairment), mean ± SD	98.2 ± 5.0	98.4 ± 4.8	t = -0.51, p = 0.604
History of TIA or stroke			χ^2^ = 7.56, p = 0.006*
Yes, n (%)	183 (33)	54 (24)	
No, n (%)	371 (67)	168 (76)	
Physical status			χ^2^ = 12.42, p = 0.000*
Healthy, n (%)	45 (8)	32 (14)	
Unhealthy, n (%)	509 (92)	190 (86)	

Abbreviations: MMSE = Mini Mental State Examination, GDS = Geriatric Depression Scale, BI = Barthel Index, ASA = American Society of Anesthesiologists score.* = significant difference between the cohort and the included subjects.

**Table 2:** Demographics and risk factors for delirium the total included sample.

	Total (N = 222)	Non-hospitalized surgery subjects (N = 182)	General practitioner subjects (N = 40)
Center			
Berlin, n (%)	67 (30)	67 (37)	0 (0)
Utrecht, n (%)	155 (70)	115 (63)	40 (100)
Male, n (%)	140 (63)	116 (63)	24 (60)
IQ, mean ± SD	105 ± 12.2	105 ± 11.5	106 ± 13.1
Age in years, mean ± SD	71.2 ± 4.9	71.2 ± 4.9	70.7 ± 4.9
Lowest quintile, cut-off, n (%)	≤67, 67 (23)		
Highest quintile, cut-off, n (%)	≥75, 68 (24)		
Alcohol misuse			
Yes, n (%)	11 (5)	11 (6)	0 (0)
No, n (%)	211 (95)	171 (94)	40 (100)
MMSE (cognitive impairment), mean ± SD	28.7 ± 1.4	28.7 ± 1.3	28.7 ± 1.5
Lowest quintile, cut-off, n (%)	≤27, 46 (16)		
Highest quintile, cut-off, n (%)	30, 96 (34)		
GDS (depression)			
Yes, n (%)	10 (5)	8 (5)	2 (5)
No, n (%)	212 (95)	174 (95)	38 (95)
BI (functional impairment), mean ± SD	98.4 ± 4.8	98.1 ± 5.2	99.4 ± 2.6
Lowest quintile, cut-off, n (%)	≤99, 50 (18)		
Highest quintile, cut-off, n (%)	100, 229 (82)		
History of TIA or stroke			
Yes, n (%)	54 (24)	51 (28)	3 (8)
No, n (%)	168 (76)	131 (72)	37 (92)
Physical status			
Healthy, n (%)	32 (14)	19 (11)	13 (33)
Unhealthy, n (%)	190 (86)	163 (89)	27 (67)

Abbreviations: MMSE = Mini Mental State Examination, GDS = Geriatric Depression Scale, BI = Barthel Index, ASA = American Society of Anesthesiologists score.

### 3.2 Models of individual risk factors

**Fig. 2:**
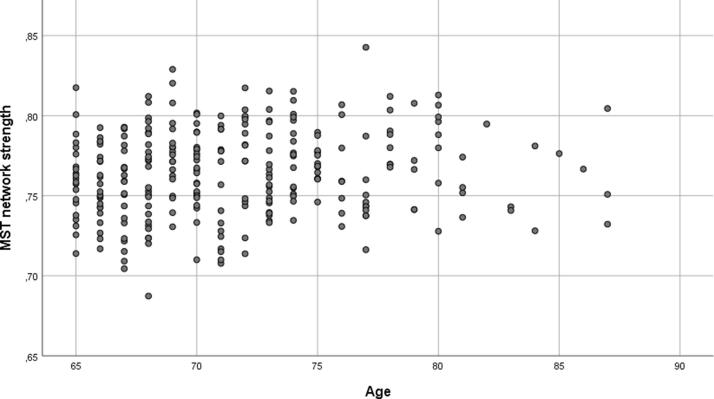
The relationship between age and global functional connectivity strength. A significant association was found between age and global functional connectivity strength. The association was independent of other risk factors for delirium, center, intelligence quotient or gender.

The results of the models on individual risk factors and the five outcome measures (i.e. global functional connectivity strength, MST diameter, MST leaf fraction, functional connectivity strength between PCC and DLPFC left and between PCC and DLPFC right) are shown in Table 3. A significant effect of age on global functional connectivity strength was found (F(4, 216) = 5.82, β = 0.178, p = 0.007, p < 0.05 after FDR correction) (Fig. 2), but in the opposite direction than expected. None of the other delirium risk factors were associated with any of the outcome measures. Rerunning our analyses while excluding the participants that were recruited via a local general practitioner revealed the same results.

**Table 3:** Results of the individual risk factors and the risk factors combined models on functional connectivity, MST diameter and MST leaf fraction.

	Functional connectivity strength			MST diameter (network efficiency)			MST leaf fraction (network integration)		
	adj. R^2^	β	Sig. (*p*)	adj. R^2^	β	Sig. (*p*)	adj. R^2^	β	Sig. (*p*)
Individual risk factors ^a^									
Age	0.081	0.178	0.007*	0.009	−0.017	0.804	0.023	0.011	0.875
Alcohol misuse	0.077	−0.023	0.726	0.008	0.068	0.323	0.025	0.081	0.233
MMSE (cognitive impairment)	0.095	0.144	0.039	0.005	0.030	0.683	0.021	0.053	0.462
GDS (depression)	0.076	−0.002	0.979	0.005	0.035	0.612	0.022	0.064	0.352
BI (functional impairment)	0.083	−0.082	0.220	0.013	−0.098	0.155	0.021	0.051	0.455
History of TIA or stroke	0.079	0.050	0.444	0.006	−0.040	0.555	0.023	0.069	0.311
ASA (physical status)	0.081	0.068	0.310	0.005	0.038	0.591	0.020	0.046	0.510
All risk factors combined ^a^	0.092			−0.004			0.026		
Age		0.183	0.007*		−0.026	0.708		−0.053	0.957
Alcohol misuse		−0.081	0.233		0.065	0.344		0.071	0.293
MMSE (cognitive impairment)		0.147	0.036		0.026	0.721		0.060	0.403
GDS (depression)		−0.019	0.779		0.003	0.967		0.076	0.285
BI (functional impairment)		−0.081	0.233		−0.097	0.176		0.082	0.248
History of TIA or stroke		0.042	0.524		−0.041	0.554		0.077	0.263
ASA (physical status)		0.067	0.326		0.039	0.584		0.027	0.700

	PCC – DLPFC left			PCC – DLPFC right		
	adj. R^2^	β	Sig. (*p*)	adj. R^2^	β	Sig. (*p*)
Individual risk factors ^a^						
Age	0.004	0.034	0.661	0.003	−0.051	0.448
Alcohol misuse	−0.001	0.000	0.995	−0.001	−0.019	0.780
MMSE (cognitiveimpairment)	−0.001	0.022	0.763	−0.002	−0.010	0.892
GDS (depression)	−0.001	−0.014	0.846	−0.001	−0.029	0.676
BI (functional impairment)	0.018	0.140	0.763	−0.001	0.034	0.620
History of TIA or stroke	0.006	0.082	0.226	0.000	0.042	0.534
ASA (physical status)	0.002	0.056	0.419	0.013	0.084	0.228
All risk factors combined ^a^	0.006			−0.015		
Age		0.031	0.651		−0.063	0.373
Alcohol misuse		−0.006	0.927		−0.025	0.715
MMSE (cognitive impairment)		0.035	0.629		−0.003	0.971
GDS (depression)		0.028	0.701		−0.023	0.752
BI (functional impairment)		0.151	0.035		0.032	0.658
History of TIA or stroke		0.080	0.251		0.025	0.725
ASA (physical status)		0.044	0.535		0.082	0.255

^a^ Models corrected for center, age (if age was not the determinant), gender and IQ. *Corrected p-value (after False Discovery Rate correction) < 0.05. Shown p-values are uncorrected for multiple testing. Abbreviations: MMSE = Mini Mental State Examination, GDS = Geriatric Depression Scale, BI = Barthel Index, TIA = transient ischemic attack, ASA = American Society of Anesthesiologists score, PCC = posterior cingulate cortex, DLPFC = dorsolateral prefrontal cortex.

### 3.3 Models of all risk factors combined

The results of the multivariable models, to test the effect of all seven risk factors on the five outcome measures are also shown in Table 3. Age was positively associated with global functional connectivity strength, independent of other risk factors, center, IQ and gender (F(10,211) = 3.195, β = 0.182*,* p = 0.008, p < 0.05 after FDR correction). The other multivariable models did not show an association with delirium risk factors. Rerunning our analyses while excluding the participants that were recruited via a local general practitioner revealed the same results.

### 3.4 Post-hoc extreme quintiles comparisons

Comparing the extreme quintiles within the continuous variables (i.e. age, cognitive impairment and functional impairment), showed an association of age on global functional connectivity strength (t(126) = 2.860, p = 0.005). Other comparisons within the extreme quintiles of the continuous variables did not show statistically significant associations.

## 4 Discussion

We tested the hypothesis that predisposing risk factors for delirium, separate or combined, are associated with delirium-related fMRI network characteristics, i.e. decreased global functional connectivity strength, decreased network efficiency, decreased network integration, altered regional functional connectivity between the PCC and the DLPFC. None of the studied predisposing risk factors for delirium affected fMRI network characteristics in the direction of disturbances observed during delirium. In this cohort of elderly subjects, age was related to an increased connectivity strength, which is an association in the opposite direction than hypothesized. No association was found between age and network efficiency or network integration. Therefore, predisposing delirium risk factors seem not to decrease connectivity strength, efficiency or integration of functional brain networks.

The finding of a positive relationship between age and global functional connectivity strength is in contrast with the existing literature, showing decreased global functional connectivity strength when older subjects were compared to younger subjects (Ferreira et al., 2016, van Montfort et al., 2019, Geerligs et al., 2015). Differences between our results and previous findings may relate to differences in methodology. Previous studies have used other network characteristics and have compared groups of younger subjects to older subjects. We studied age as a continuous variable and included a sample with less contrast, that only contained subjects of 65 years and above, and used MST backbone network characteristics. The MST uses only the backbone network to calculate global functional connectivity strength resulting in connections with a higher signal-to-noise ratio, which may partly explain the differences between our work and previous studies. It should however be noted that the effect size of the association of age with global functional connectivity strength was small, i.e. although the relationship was statistically significant, only a limited proportion of variation was explained by the model. In addition, as patients (especially the oldest part of our sample) should be in a relatively healthy general condition to be scheduled for elective surgery, this finding might be influenced by a cohort effect. Therefore, we cannot draw firm conclusions on the relationship between age and global functional connectivity strength in elderly based on our results.

This study is the first to empirically investigate the association between predisposing risk factors for delirium and delirium-related fMRI network characteristics in the same study population. We used robust methods and included a large number of participants in this multicenter study. However, the selection of the study population may be considered as a limitation of the study. As the selection was performed from a relatively uniform, relatively healthy elderly population, a strong contrast between subjects experiencing risk factors versus subjects not experiencing risk factors was lacking. If the part of the group with delirium risk factors would have been compared to a healthy young group, the results might have differed. Furthermore, the selection of predisposing risk factors was based on an influential review (Inouye et al., 2014), and not on more recent systematic reviews, as these latter publications included prediction models instead of etiologic models. Prediction models yield predictors that may not necessarily play a role in the pathophysiology of delirium, an example is ‘urgent admission’. The interpretation of our study is limited to the included predisposing risk factors. Another limitation is the exclusion of a considerable part of our sample due to strict motion correction. In particular, frail elderly may have had problems with laying completely still and may therefore have been excluded from the study. This may have resulted in a selection of a healthier and younger part of the cohort. As motion during fMRI measurement can induce systematical bias, we were forced to perform this rigorous motion correction (Ciric et al., 2017, Power et al., 2015, Power et al., 2012, Pruim et al., 2015). Another important limitation of the study is that information on medication use (e.g. psychotropic or antiepileptic drugs) of the subjects during the fMRI measurements was not available. We therefore cannot exclude effects of possible medication on the fMRI measurements. However, the subjects were derived from a relatively healthy non-hospitalized population. Further, we focused on fMRI network characteristics that are altered during delirium, and did not evaluate the relationship between delirium risk factors and all possible fMRI (network) characteristics or used seed-based analyses to focus on unexplored regional connections that are altered in patients at risk for delirium. It could therefore be that functional brain impairments related to vulnerability to delirium are represented in other fMRI outcomes. In addition, the structural network was not evaluated in this study. The risk profile for delirium might as well be reflected in structural network abnormalities, as we found in a recent review and meta-analysis (van Montfort et al., 2019).

Our findings are in line with our recent study investigating the association between predisposing risk for delirium and delirium-related neurophysiological alterations using EEG (van Montfort et al., 2020). Taken together, these two studies did not find (strong) evidence for the hypothesis that predisposing risk for delirium is related to the same brain network disturbances as are observed in delirium. Nevertheless, an alternative hypothesis may be that predisposition for delirium is defined by other functional brain (network) characteristics than the profile of delirium itself. In other words, it could be that other parameters reflect a predisposing state than those that are altered during delirium. On the other hand, it could be that predisposing risk for delirium is solely related to structural network abnormalities (Kyeong et al., 2018, van Montfort et al., 2019), while precipitating risk factors and the fluctuating nature of delirium itself may be characterized by functional network impairments (Blain-Moreas et al., 2017, Lee, 2013, Maestu et al., 2010, Mashour et al., 2018, Numan et al., 2017, van Dellen et al., 2014, van Montfort et al., 2018). Predisposing and precipitating risk factors are expected to cause delirium in a complex interaction (Inouye et al., 2014, Maldonado, 2018, Sanders, 2011, Young, 2017). The scope of the current work was to test the hypothesis that predisposing risk for delirium is reflected in the functional brain network. Future work should elucidate the predisposing risk of delirium in relation to precipitating events and the occurrence of delirium itself, which are currently subject of study.

## 5 Conclusions

This study was the first to empirically evaluate the hypothesis of functional network impairments as biological pathways underlying vulnerability for delirium, using fMRI. None of the predisposing risk factors for delirium was associated with decreased global functional connectivity strength, network efficiency, network integration or the regional functional connectivity between the posterior cingulate and the dorsolateral prefrontal cortex. We therefore conclude that predisposition for delirium is not consistently associated to delirium-related functional network alterations, as studied with fMRI.

## CRediT authorship contribution statement

**S.J.T. Montfort:** Conceptualization, Formal analysis, Investigation, Writing - original draft. **A.J.C. Slooter:** Conceptualization, Resources, Writing - review & editing, Supervision. **I.M.J. Kant:** Investigation, Writing - review & editing. **R.R. Leur:** Investigation, Software, Writing - review & editing. **C. Spies:** Resources. **J. Bresser:** Validation. **T.D. Witkamp:** Validation. **J. Hendrikse:** Resources, Writing - review & editing, Supervision. **E. Dellen:** Conceptualization, Writing - review & editing, Supervision.

## Declaration of Competing Interest

The authors declare that they have no known competing financial interests or personal relationships that could have appeared to influence the work reported in this paper.

## References

[b0005] Aertsen A.M., Gerstein G.L., Habib M.K., Palm G. (1989). Dynamics of neuronal firing correlation: modulation of “effective connectivity”. J. Neurophysiol..

[b0010] American Psychiatric Association, 2013. Diagnostic and statistical manual of mental disorders, fifth ed., Washington, DC.

[b0015] Aronson W.L., McAuliffe M.S., Miller K. (2003). Variability in the American Society of Anesthesiologists Physical Status Classification Scale. AANA J..

[b0020] Babor, T., Higgins-Biddle, J., Saunders, J., 2001. AUDIT: the alcohol use disorders identification test: guidelines for use in primary health care.

[b0025] Bassett D.S., Sporns O. (2017). Network neuroscience. Nat. Neurosci..

[b0030] Benjamini Y., Hochberg Y. (1995). Controlling the false discovery rate: a practical and powerful approach to multiple testing. J. R. Stat. Soc. Ser. B.

[b0035] Birn R.M., Molloy E.K., Patriat R., Parker T., Meier T.B., Kirk G.R., Nair V.A., Meyerand M.E., Prabhakaran V. (2013). The effect of scan length on the reliability of resting-state fMRI connectivity estimates. Neuroimage.

[bib312] Blain-Moreas S. (2017). Network efficiency and posterior alpha patterns are markers of recovery from general anesthesia: a high-density electroencephalography study in healthy volunteers. Front. Hum. Neurosci..

[b0040] Bohn M.J., Babor T.F., Kranzler H.R. (1995). The Alcohol Use Disorders Identification Test (AUDIT): validation of a screening instrument for use in medical settings. J. Stud. Alcohol.

[b0045] Choi S.H., Lee H., Chung T.S., Park K.M., Jung Y.C., Kim S.I., Kim J.J. (2012). Neural network functional connectivity during and after an episode of delirium. Am. J. Psychiatry.

[b0050] Ciric R., Wolf D.H., Power J.D., Roalf D.R., Baum G.L., Ruparel K., Shinohara R.T., Elliott M.A., Eickhoff S.B., Davatzikos C., Gur R.C., Gur R.E., Bassett D.S., Satterthwaite T.D. (2017). Benchmarking of participant-level confound regression strategies for the control of motion artifact in studies of functional connectivity. Neuroimage.

[b0055] Collin C., Wade D.T., Davies S., Horne V. (1988). The Barthel ADL Index: a reliability study. Int. Disabil. Stud..

[b0060] Ferreira L.K., Regina A.C.B., Kovacevic N., Martin M.D.G.M., Santos P.P., Carneiro C.D.G., Kerr D.S., Amaro E., Mcintosh A.R., Busatto G.F. (2016). Aging effects on whole-brain functional connectivity in adults free of cognitive and psychiatric disorders. Cereb. Cortex.

[b0065] Folstein M., Anthony J.C., Parhad I., Duffy B., Gruenberg E.M. (1985). The meaning of cognitive impairment in the elderly. J. Am. Geriatr. Soc..

[b0070] Geerligs, L., Rubinov, M., Cam-Can, Henson, R.N., 2015. State and Trait Components of Functional Connectivity: Individual Differences Vary with Mental State. J. Neurosci. 35, 13949–61, doi: 10.1523/JNEUROSCI.1324-15.2015. 10.1523/JNEUROSCI.1324-15.2015 PMC4604231 26468196

[b0075] Genovese C.R., Lazar N.A., Nichols T. (2002). Thresholding of statistical maps in functional neuroimaging using the false discovery rate. Neuroimage.

[b0080] Inouye S.K., Westendorp R.G., Saczynski J.S. (2014). Delirium in elderly people. Lancet.

[b0085] Jenkinson M., Bannister P., Brady M., Smith S. (2002). Improved optimization for the robust and accurate linear registration and motion correction of brain images. Neuroimage.

[b0090] Jenkinson M., Beckmann C.F., Behrens T.E.J., Woolrich M.W., Smith S.M. (2012). FSL. Neuroimage.

[b0095] Jenkinson M., Smith S. (2001). A global optimisation method for robust affine registration of brain images. Med. Image Anal..

[b0100] Kruskal J.B. (1956). On the shortest spanning subtree of a graph and the traveling salesman problem. Proc. Am. Math. Soc..

[b9005] Kyeong S., Shin J.E., Yang K.H., Lee W.S., Chung T.-S., Kim J.-J. (2018 May 15). Neural predisposing factors of postoperative delirium in elderly patients with femoral neck fracture. Sci. Rep..

[bib314] Lee H. (2013). Reconfiguration of network hub structure after propofol-induced unconsciousness. Anesthesiology.

[b0105] Lehrl S., Triebig G., Fischer B. (1995). Multiple choice vocabulary test MWT as a valid and short test to estimate premorbid intelligence. Acta Neurol. Scand..

[b0110] Lübke N., Meinck M., Von Renteln-Kruse W. (2004). The Barthel Index in geriatrics. A context analysis for the Hamburg Classification Manual. Z. Gerontol. Geriatr..

[bib313] Maestu F. (2010). Principles of recovery from traumatic brain injury: reorganization of functional networks. NeuroImage.

[b0115] Mahoney F.I., Barthel D.W. (1965). functional evaluation: the Barthel index. Md. State Med. J..

[b0120] Maldonado J.R. (2018). Delirium pathophysiology: an updated hypothesis of the etiology of acute brain failure. Int. J. Geriatr. Psychiatry.

[b0125] Marcantonio E.R. (2017). Delirium in hospitalized older adults. N. Engl. J. Med..

[bib315] Mashour G.A. (2018). Neural correlates of unconsciousness in large-scale brain networks. Trends. Neurosci..

[b0130] Mulder J., Bouma J.M., Schmand B. (2012). Handboek neuropsychologische diagnostiek.

[b0135] Numan T., Slooter A.J.C., van der Kooi A.W., Hoekman A.M.L., Suyker W.J.L., Stam C.J., van Dellen E. (2017). Functional connectivity and network analysis during hypoactive delirium and recovery from anesthesia. Clin. Neurophysiol..

[b0140] Oh J., Shin J.E., Yang K.H., Kyeong S., Lee W.S., Chung T.-S., Kim J.-J. (2019). Cortical and subcortical changes in resting-state functional connectivity before and during an episode of postoperative delirium. Aust. New Zeal. J. Psychiatry.

[b0145] Otte W.M., van Diessen E., Paul S., Ramaswamy R., Subramanyam Rallabandi V.P., Stam C.J., Roy P.K. (2015). Aging alterations in whole-brain networks during adulthood mapped with the minimum spanning tree indices: the interplay of density, connectivity cost and life-time trajectory. Neuroimage.

[b0150] Owens W.D., Felts J.A., Spitznagel E.L. (1978). ASA physical status classifications: a study of consistency of ratings. Anesthesiology.

[b0155] Power J.D., Barnes K.A., Snyder A.Z., Schlaggar B.L., Petersen S.E. (2012). Spurious but systematic correlations in functional connectivity MRI networks arise from subject motion. Neuroimage.

[b0160] Power J.D., Cohen A.L., Nelson S.M., Wig G.S., Barnes K.A., Church J.A., Vogel A.C., Laumann T.O., Miezin F.M., Schlaggar B.L., Petersen S.E. (2011). Functional network organization of the human brain. Neuron.

[b0165] Power J.D., Mitra A., Laumann T.O., Snyder A.Z., Schlaggar B.L., Petersen S.E. (2014). Methods to detect, characterize, and remove motion artifact in resting state fMRI. Neuroimage.

[b0170] Power J.D., Schlaggar B.L., Petersen S.E. (2015). Recent progress and outstanding issued in motion correction resting state fmri. Neuroimage.

[b0175] Pruim R.H.R., Mennes M., Buitelaar J.K., Beckmann C.F. (2015). Evaluation of ICA-AROMA and alternative strategies for motion artifact removal in resting state fMRI. Neuroimage.

[b0180] Reinert D.F., Allen J.P. (2007). The alcohol use disorders identification test: an update of research findings. Alcohol. Clin. Exp. Res..

[b0185] Sanders R.D. (2011). Hypothesis for the pathophysiology of delirium: role of baseline brain network connectivity and changes in inhibitory tone. Med. Hypotheses.

[b0190] Sankar A., Johnson S.R., Beattie W.S., Tait G., Wijeysundera D.N. (2014). Reliability of the American Society of Anesthesiologists physical status scale in clinical practice. Br. J. Anaesth..

[b0195] Satterthwaite T.D., Elliott M.A., Gerraty R.T., Ruparel K., Loughead J., Calkins M.E., Eickhoff S.B., Hakonarson H., Gur R.C., Gur R.E., Wolf D.H. (2013). An improved framework for confound regression and filtering for control of motion artifact in the preprocessing of resting-state functional connectivity data. Neuroimage.

[b0200] Slooter, A., Otte, W., Devlin, J., Arora, R., Bleck, T., Claassen, J., Duprey, M., Ely, E., Kaplan, P., Latronico, N., Morandi, A., Neufeld, K., Sharshar, T., MacLullich, A., Stevens, R., 2020. Updated nomenclature of delirium and acute encephalopathy. Statements endorsed by the American Academy of Neurology (AAN), American Delirium Society (ADS), European Academy of Neurology (EAN), European Delirium Association (EDA), European Geriatric Medici. Intensive Care Med.

[b0205] Smith S.M. (2002). Fast robust automated brain extraction. Hum. Brain Mapp..

[b0210] Smith S.M., Jenkinson M., Woolrich M.W., Beckmann C.F., Behrens T.E.J., Johansen-Berg H., Bannister P.R., De Luca M., Drobnjak I., Flitney D.E., Niazy R.K., Saunders J., Vickers J., Zhang Y., De Stefano N., Brady J.M., Matthews P.M. (2004). Advances in functional and structural MR image analysis and implementation as FSL. NeuroImage..

[b0215] Stam C.J., Tewarie P., Van Dellen E., van Straaten E.C.W., Hillebrand A., Van Mieghem P. (2014). The trees and the forest: Characterization of complex brain networks with minimum spanning trees. Int. J. Psychophysiol..

[b0220] Stumme J., Jockwitz C., Hoffstaedter F., Amunts K., Caspers S. (2020). Functional network reorganization in older adults: Graph-theoretical analyses of age, cognition and sex. Neuroimage.

[b0225] Tewarie P., van Dellen E., Hillebrand A., Stam C.J. (2015). The minimum spanning tree: an unbiased method for brain network analysis. Neuroimage.

[b0230] van Dellen E., Bohlken M.M., Draaisma L., Tewarie P.K., van Lutterveld R., Mandl R., Stam C.J., Sommer I.E. (2016). Structural brain network disturbances in the psychosis spectrum. Schizophr. Bull..

[b0235] van Dellen E., Sommer I.E., Bohlken M.M., Tewarie P., Draaisma L., Zalesky A., Di Biase M., Brown J.A., Douw L., Otte W.M., Mandl R.C.W., Stam C.J. (2018). Minimum spanning tree analysis of the human connectome. Hum. Brain Mapp..

[b0240] van Dellen E., van der Kooi A.W., Numan T., Koek H.L., Klijn F.A.M., Buijsrogge M.P., Stam C.J., Slooter A.J.C. (2014). Decreased functional connectivity and disturbed directionality of information flow in the electroencephalography of intensive care unit patients with delirium after cardiac surgery. Anesthesiology.

[b0245] van den Heuvel M.P., Hulshoff Pol H.E. (2010). Exploring the brain network: a review on resting-state fMRI functional connectivity. Eur. Neuropsychopharmacol..

[b0250] van Diessen E., Numan T., van Dellen E., van der Kooi A.W., Boersma M., Hofman D., van Lutterveld R., van Dijk B.W., van Straaten E.C.W., Hillebrand A., Stam C.J. (2015). Opportunities and methodological challenges in EEG and MEG resting state functional brain network research. Clin. Neurophysiol..

[b0260] van Montfort, S., van Dellen, E., Wattel, L., Kant, I., Numan, T., Stam, C., Slooter, A., 2020. Predisposition for delirium and EEG characteristics. Clin. Neurophysiol. 10.1016/j.clinph.2020.01.023 32199395

[b0255] van Montfort S.J.T., van Dellen E., Stam C., Ahmad A., Mentink L., Kraan C., Zalesky A., Slooter A. (2019). Brain network disintegration as a final common pathway for delirium: a systematic review. NeuroImage Clin..

[b0270] van Montfort S.J.T., van Dellen E., van den Bosch A.M.R., Otte W.M., Schutte M.J.L., Choi S.-H., Chung T.-S., Kyeong S., Slooter A.J.C., Kim J.-J. (2018). Resting-state fMRI reveals network disintegration during delirium. NeuroImage Clin..

[b0275] Wardlaw, J.M., Smith, E.E., Biessels, G.J., Cordonnier, C., Fazekas, F., Frayne, R., Lindley, R.I., O’Brien, J.T., Barkhof, F., Benavente, O.R., Black, S.E., Brayne, C., Breteler, M., Chabriat, H., DeCarli, C., de Leeuw, F.-E., Doubal, F., Duering, M., Fox, N.C., Greenberg, S., Hachinski, V., Kilimann, I., Mok, V., Oostenbrugge, R. van, Pantoni, L., Speck, O., Stephan, B.C.M., Teipel, S., Viswanathan, A., Werring, D., Chen, C., Smith, C., van Buchem, M., Norrving, B., Gorelick, P.B., Dichgans, M., STandards for ReportIng Vascular changes on nEuroimaging (STRIVE v1), 2013. Neuroimaging standards for research into small vessel disease and its contribution to ageing and neurodegeneration. Lancet Neurol. 12, 822–838, doi: 10.1016/S1474-4422(13)70124-8. 10.1016/S1474-4422(13)70124-8 PMC3714437 23867200

[b0280] Winterer, G., Androsova, G., Bender, O., Boraschi, D., Borchers, F., Dschietzig, T.B., Feinkohl, I., Fletcher, P., Gallinat, J., Hadzidiakos, D., Haynes, J.D., Heppner, F., Hetzer, S., Hendrikse, J., Ittermann, B., Kant, I.M.J., Kraft, A., Krannich, A., Krause, R., Kühn, S., Lachmann, G., van Montfort, S.J.T., Müller, A., Nürnberg, P., Ofosu, K., Pietsch, M., Pischon, T., Preller, J., Renzulli, E., Scheurer, K., Schneider, R., Slooter, A.J.C., Spies, C., Stamatakis, E., Volk, H.D., Weber, S., Wolf, A., Yürek, F., Zacharias, N., 2018. Personalized risk prediction of postoperative cognitive impairment - rationale for the EU-funded BioCog project. Eur. Psychiatry, doi: 10.1016/j.eurpsy.2017.10.004. 10.1016/j.eurpsy.2017.10.004 29398565

[b0285] Woolrich M.W., Jbabdi S., Patenaude B., Chappell M., Makni S., Behrens T., Beckmann C., Jenkinson M., Smith S.M. (2009). Bayesian analysis of neuroimaging data in FSL. Neuroimage.

[b0290] Yesavage J.A., Brink T.L., Rose T.L., Lum O., Huang V., Adey M., Leirer V.O. (1982). Development and validation of a geriatric depression screening scale: a preliminary report. J. Psychiatr. Res..

[b0295] Yesavage J.A., Sheikh J.I. (1986). Geriatric Depression Scale (GDS): recent evidence and development of a shorter version. Clin. Gerontol..

[b0300] Young J.W.S. (2017). The network model of delirium. Med. Hypotheses.

[b0305] Zaal I.J., Devlin J.W., Peelen L.M., Slooter A.J.C. (2015). A systematic review of risk factors for delirium in the ICU*. Crit. Care Med..

[b0310] Zhang Y., Brady M., Smith S. (2001). Segmentation of brain MR images through a hidden Markov random field model and the expectation-maximization algorithm. IEEE Trans. Med. Imaging.

