# Supplementary material for: fMRI network correlates of predisposing risk factors for delirium: A cross-sectional study
Source: Neuroimage Clin. 2020 Jul 15;27:102347. doi: 10.1016/j.nicl.2020.102347 (PMC7394743; doi:10.1016/j.nicl.2020.102347)
Supplement: Supplementary data 1 [file mmc1.docx]

**Supplementary Information**


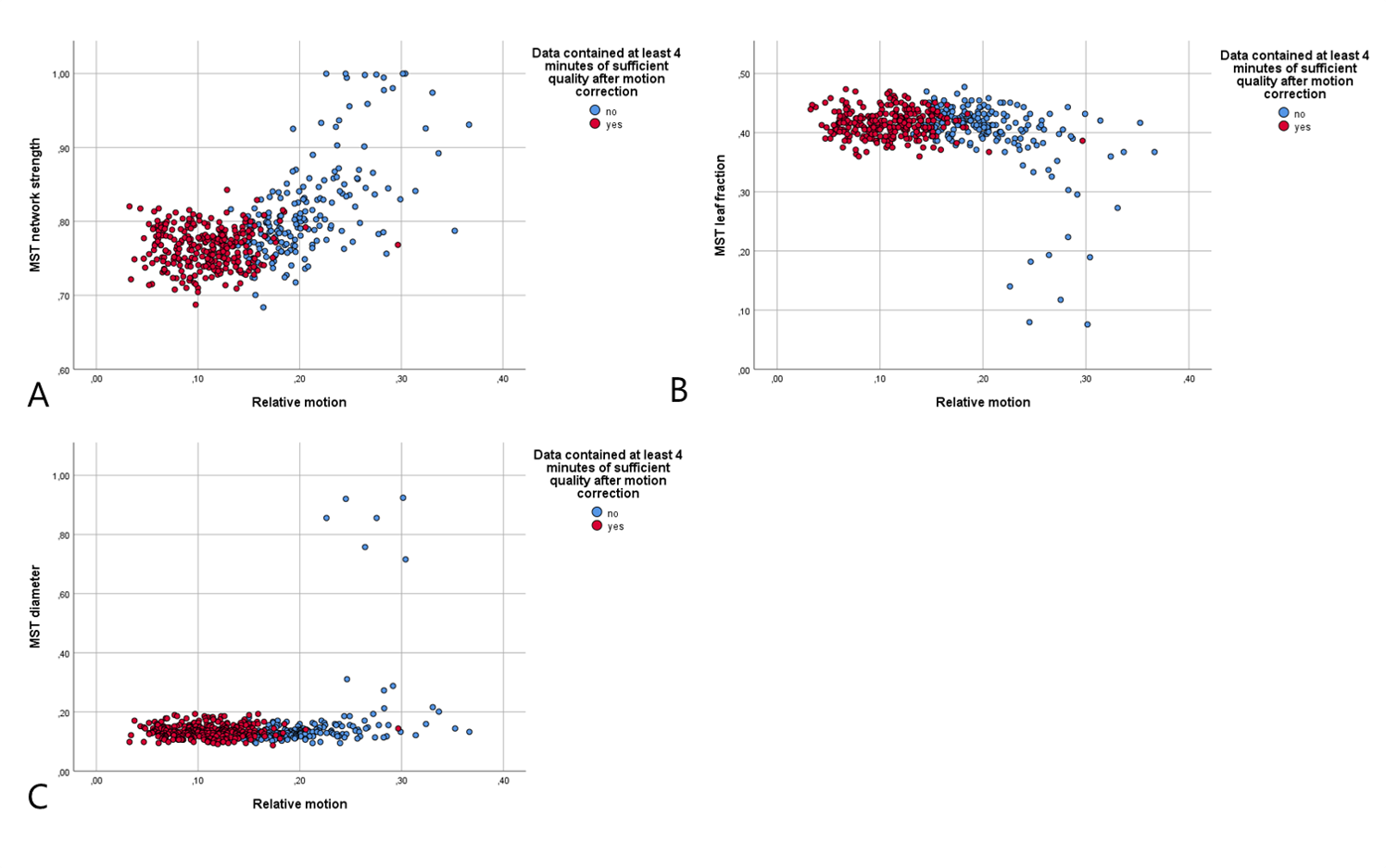


**Figure S1.** Scatterplots of network characteristics (functional connectivity strength, diameter and leaf fraction) by relative motion in the total sample. In scatterplots (A), (B) and (C) the total eligible sample is depicted (N = 554). The red dotes represent the subjects with at least 4 minutes data of sufficient quality after motion correction (the included sample of this study (N = 222)) and the blue dotes the subjects with less than 4 minutes data of sufficient quality after motion correction (excluded from this study). In the total cohort (N = 554) significant Pearson’s correlations were found between relative motion and functional connectivity strength (r = 0.59, p < 0.000), relative motion and MST diameter (r = 0.26, p < 0.000) or relative motion and MST leaf fraction (r = -0.33, p < 0.000).


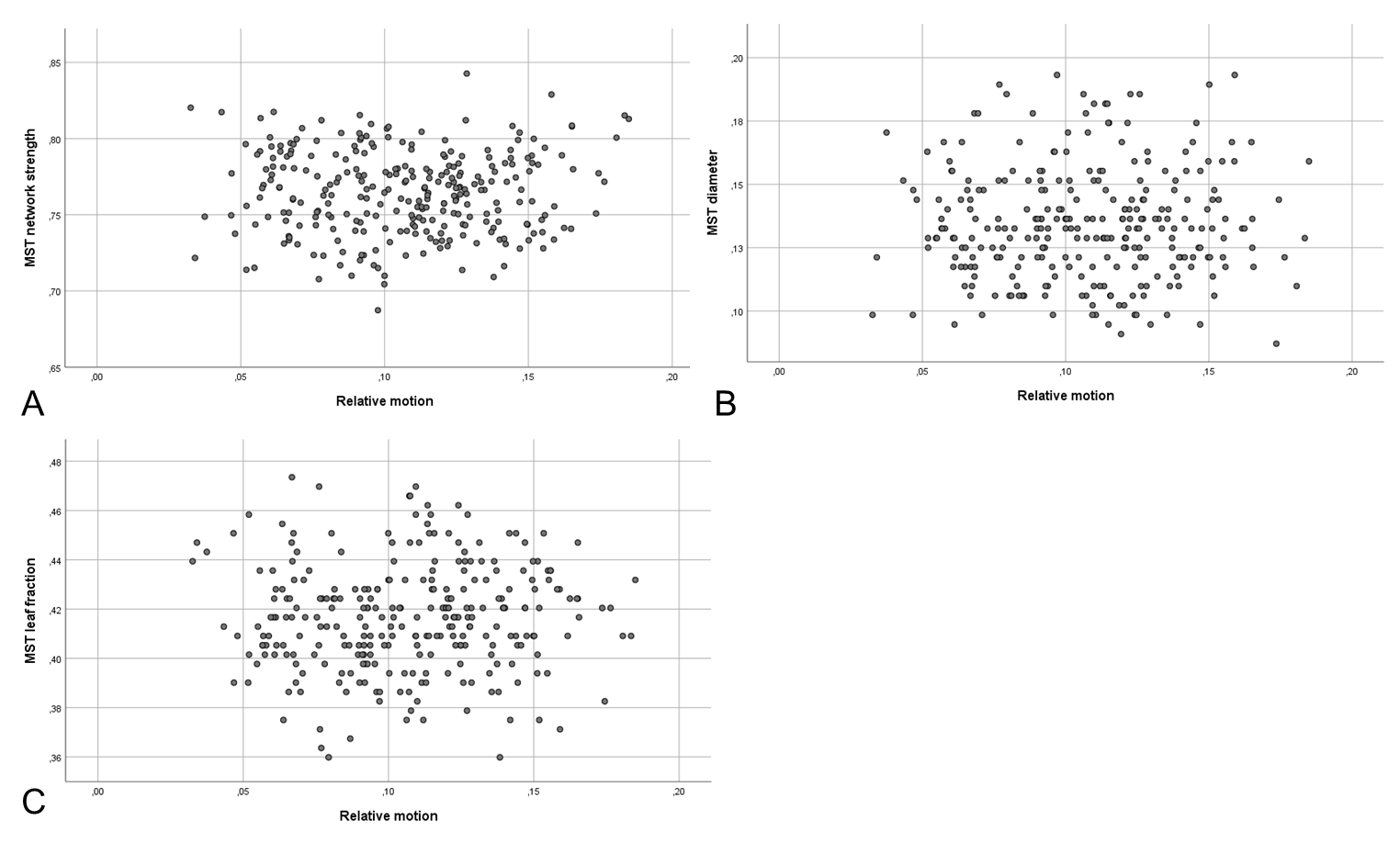


**Figure S2.** Scatterplots of network characteristics (functional connectivity strength, diameter and leaf fraction) by relative motion in the included sample. Scatterplots (A), (B) and (C) show the network characteristics by relative motion after motion correction of the included subjects in this study (N = 222). In the included sample, no significant Pearson’s correlations were found between relative motion and functional connectivity strength (r = 0.02, p = 0.746), relative motion and MST diameter (r = 0.02, p = 0.685) or relative motion and MST leaf fraction (r = -0.12, p = 0.844).
